# Cross-tissue transcriptome-wide association identify novel T1D susceptibility genes and drug candidates

**DOI:** 10.3389/fimmu.2025.1735004

**Published:** 2026-01-21

**Authors:** Yiming Liu, Yu Cao, Yaohui Jiang

**Affiliations:** 1 Department of Cardiology, National Cardiovascular Disease Regional Center for Anhui, The First Affiliated Hospital of Anhui Medical University, Hefei, Anhui, China; 2 Department of Endocrinology, The Third Xiangya Hospital of Central South University, Science and Education Building, Changsha, Hunan, China; 3 Department of Cardiology, Fuwai Central China Cardiovascular Hospital, Henan Key Laboratory of Coronary Heart Disease Control & Prevention, Central China Fuwai Hospital of Zhengzhou University, Zhengzhou, Henan, China

**Keywords:** colocalization, cross-tissue TWAS, Mendelian randomization, single-cell sequencing, type 1 diabetes

## Abstract

**Background:**

The genetic mechanisms underlying type 1 diabetes (T1D) remain incompletely understood, limiting the development of targeted therapies.

**Methods:**

We performed an integrative genetic analysis to identify T1D susceptibility genes and therapeutic targets. This included a cross-tissue transcriptome-wide association study (TWAS) to pinpoint genes with genetically predicted expression associated with T1D risk, followed by Mendelian randomization to infer causality. Identified genes were further characterized through pathway, cell-type enrichment, drug prediction, molecular docking, and phenome-wide association studies.

**Results:**

We identified ten genes associated with T1D risk, seven of which (ELK4, PHACTR4, MAST2, ST7L, C1orf216, SULT1A2, and WFS1) are novel candidates in this context. Three genes (ELK4, SULT1A2, and WFS1) were prioritized as druggable targets, with COMPOUND 5G and DCLK1-IN-1 emerging as potential therapeutic agents through computational analyses.

**Conclusion:**

Our study reveals novel genetic associations and immune-related pathways in T1D pathogenesis, and proposes specific genes and compounds as promising focal points for future mechanistic and therapeutic exploration.

## 1 Introduction

The global incidence of type 1 diabetes (T1D) has been increasing steadily for several decades (1). The disease is characterized by an autoimmune response that leads to immune cell infiltration of pancreatic islets and the subsequent destruction of insulin-producing β-cells (2, 3). At present, insulin replacement therapy remains the primary treatment modality for preventing life-threatening outcomes in patients with T1D. However, the underlying pathogenic mechanisms of T1D are not yet fully understood, underscoring the need for comprehensive approaches to identify potential therapeutic targets and to elucidate disease pathogenesis for prevention and treatment.

Over the past few decades, numerous studies have sought to identify genes involved in T1D pathogenesis. To date, more than 60 candidate susceptibility loci have been reported (4). In population-level genome-wide association studies (GWAS) and functional studies, genes such as insulin (5), CTLA-4 (6), PTPN22 (7), and IL2RA (interleukin 2 receptor α) (8) have also been reported as T1D susceptibility genes. Although GWAS benefit from large-scale datasets, they are limited in their ability to explain the biological functions of variants located in non-coding regions. To address this limitation, transcriptome-wide association studies (TWAS) incorporating gene expression imputation have been developed. TWAS integrates expression quantitative trait loci (eQTL) data with GWAS summary statistics to identify susceptibility genes whose genetically predicted expression is associated with disease risk. By aggregating multiple variants into a single functional gene unit (9), TWAS reduces the burden of multiple testing and enables direct prioritization of candidate genes. As a powerful gene-based approach, TWAS has been successfully applied to elucidate the genetic architecture of a wide range of complex traits. However, most existing TWAS studies calculate genetic expression matrices separately for each tissue (10–13), which may overlook shared local regulation of gene expression between different tissues. There is evidence that eQTLs with large effects can regulate gene expression in multiple tissues (14). Recently, multi-tissue eQTL panels (15), including the S-MultiXcan model and sparse Canonical Correlation Analysis–Aggregated Cauchy Association Test(sCCA-ACAT), have shown stronger statistical power in TWAS by combining shared genetic features in gene expression regulation. Therefore, in our study, we incorporated multi-tissue eQTL panels into our TWAS analysis. TWAS has successfully provided new insights into the pathogenesis and prioritized pathogenic genes for various diseases and is one of the frontier methods for studying the triangular mechanism between genetic variation, gene expression, and phenotype (10, 16–18).

**Figure 1:**
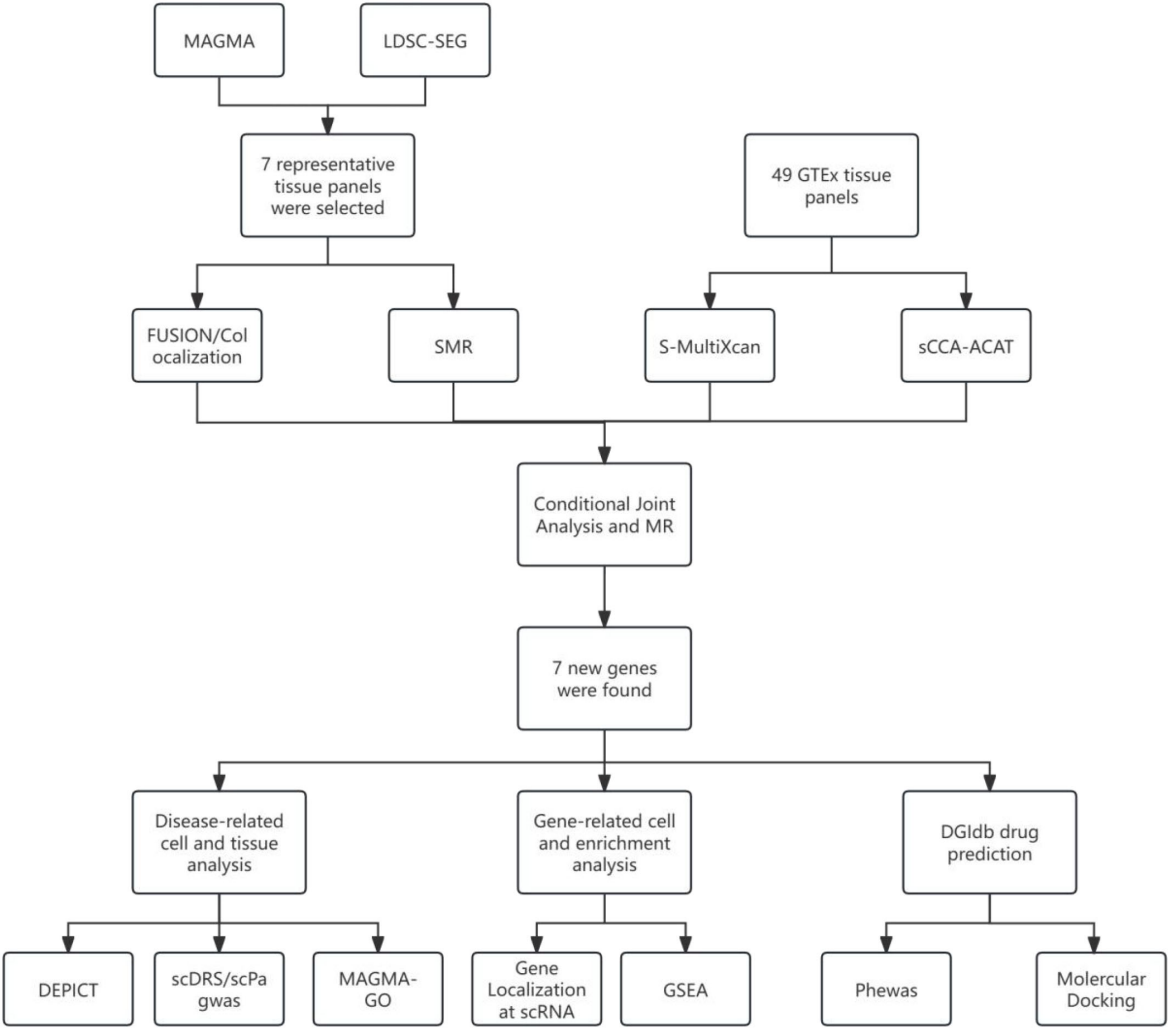
Summary of study design.

In this study, we conducted a comprehensive TWAS of T1D using publicly available GWAS summary statistics derived from individuals of European ancestry. T1D-related tissues were prioritized using MAGMA and LDSC-SEG analyses based on GWAS data from the GWAS Catalog (GCST90014023). Guided by MAGMA tissue-enrichment results, seven representative tissue panels from the Genotype-Tissue Expression (GTEx) Project (version 8) were selected. By integrating TWAS, summary-based Mendelian randomization (SMR), S-MultiXcan, and sCCA-ACAT analyses, we identified 38 genes significantly associated with T1D. Conditional and joint analyses, along with Mendelian randomization, were subsequently performed to validate robust T1D-associated markers. Tissue- and cell-type enrichment analyses using DEPICT and MAGMA-GO revealed that T1D pathogenesis is closely linked to immune responses in peripheral blood. Furthermore, single-cell transcriptomic analyses demonstrated distinct cell type–specific expression patterns for multiple candidate genes, as well as their potential functional roles as indicated by single-gene gene set enrichment analysis (GSEA). Using the Drug–Gene Interaction Database (DGIdb), three druggable genes significantly associated with T1D were identified, along with six corresponding drug–gene interaction pairs. To further explore pleiotropic effects and potential adverse drug reactions, phenome-wide association studies (PheWAS) were conducted for three SNPs linked to SULT1A2, WFS1, and ELK4. Finally, molecular docking analyses revealed stable protein–ligand interactions, supporting the therapeutic potential of these candidate targets. The overall study workflow is summarized in **Figure 1**.

## 2 Materials and methods

### 2.1 Datasets

#### 2.1.1 GWAS data

The analysis used (1) genome-wide summary statistics from the GWAS of T1D by Chiou J et al. (19), (2) 9 SNP weight sets from 3 separate transcriptomic reference samples GTEx v8, NTR (Netherlands Twin Register) and YFS (Young Finns Study) (20, 21), and (3) the 1000 Genomes Project linkage disequilibrium (LD) reference. Initially, we obtained the GWAS data of T1D from 520,580 European participants, including 18,942 cases and 501,638 controls, along with the T1D summary statistics from deCODE Genetics summary data, which contains genetic susceptibility information to T1D for 59,999,551 HapMap3 SNPs from datasets of Iceland and the UK Biobank.

#### 2.1.2 RNA-seq data

RNA-sequencing data for GSE123658 were retrieved from the Gene Expression Omnibus (GEO; http://www.ncbi.nlm.nih.gov/geo/) (22). Raw counts were normalized to counts per million (CPM) using edgeR (23), followed by voom transformation (24) and analysis with the Limma package (25) in R. The data were generated on the GPL18573 Illumina NextSeq 500 platform (Homo sapiens) and included 82 samples, comprising 39 type 1 diabetes (T1D) cases and 43 healthy controls.

### 2.2 Cell type enrichment and tissue expression analysis

#### 2.2.1 DEPICT analysis

DEPICT, an analytical framework that utilizes genome-wide association study (GWAS) summary statistics, was employed to identify candidate genes within significant loci and to assess tissue-specific enrichment patterns. In this study, we applied DEPICT (version v1_rel194) using its standard settings. The summary statistics for type 1 diabetes (T1D) were evaluated against a genetic background constructed from SNPsnap reference data based on the 1000 Genomes Project Phase 3 (26, 27), allowing for systematic gene prioritization across the associated genomic regions.

#### 2.2.2 MAGMA analysis

For gene-set and tissue enrichment analyses, we employed the MAGMA algorithm integrated within the FUMA platform (version 1.3.7) to examine 54 tissue categories using RNA-seq profiles from GTEx v8. The MAGMA gene-property analysis was conducted to estimate the mean expression level of each gene in individual tissues while adjusting for its overall expression across all tissues, applying a one-sided test. Multiple-testing correction was performed using the Bonferroni method, with statistical significance defined as p < 0.05/54. This approach evaluates the relationship between tissue-specific gene expression and GWAS-derived genetic associations. Comprehensive methodological details for the MAGMA gene-property framework are available in earlier reports (28, 29).

#### 2.2.3 LDSC-SEG

To identify the most relevant tissues for the transcriptome-wide association study (TWAS), we performed a tissue-specific heritability enrichment analysis using linkage disequilibrium (LD) score regression with the LDSC-SEG approach (29). Data on gene expression and epigenetic features—such as DNase I hypersensitivity sites, histone acetylation, and histone methylation—were obtained from the resource described by Finucane et al. (30). Tissues exhibiting a regression coefficient p-value below 0.05 were considered significantly enriched and subsequently included in the TWAS analysis.

### 2.3 TWAS FUSION and colocalization

FUSION is a computational framework designed for transcriptome-wide (TWAS) and regulome-wide association studies (RWAS). It constructs predictive models that capture the genetically regulated components of molecular or functional phenotypes, which are subsequently integrated with GWAS summary statistics to pinpoint loci associated with disease risk. In the present study, TWAS was conducted following the default parameters of the FUSION protocol (9). Critically, to account for the varying precision of gene expression prediction across genes, FUSION’s association test statistic incorporates a weighting scheme based on the prediction performance (R2) of each expression model. This adjustment effectively down-weights associations for genes with poorly predicted expression, which is mathematically equivalent to performing the test with an adjusted effective sample size. To determine whether the same causal variants underlie both T1D and gene expression, we performed Bayesian colocalization analysis (31). Implemented within the FUSION pipeline, this analysis estimated the posterior probability that a single variant was jointly associated with T1D and tissue-specific gene expression. A posterior probability greater than 0.7 was interpreted as strong evidence supporting colocalization.

### 2.4 Joint TWAS across multiple tissues

Expression quantitative trait loci (eQTL) data were integrated with GWAS summary statistics through the S-MultiXcan framework (32), implemented within the Complex Traits Genetics Virtual Lab (CTG-VL). This method applies multivariate regression of phenotypic values on genetically predicted gene expression across multiple tissues, thereby incorporating information from eQTL resources. For analyses confined to a single tissue, S-PrediXcan was used, whereas joint associations were assessed with a strategy analogous to conditional and joint multiple-SNP analysis (33).

Because a tissue-specific reference panel for type 1 diabetes (T1D) was unavailable, we adopted the recommended approach of utilizing all GTEx reference panels in the TWAS (34). To enhance statistical power, a sparse canonical correlation analysis (sCCA)-based TWAS was additionally performed using cross-tissue reference models provided by the FUSION repository. Finally, single-tissue and sCCA-TWAS results were combined using the aggregate Cauchy association test (ACAT). Further methodological details are available in earlier publications (35).

### 2.5 SMR analysis

To pinpoint genes with potential causal effects on type 1 diabetes (T1D), we conducted summary-data–based Mendelian randomization (SMR) analyses, prioritizing genes with functional relevance within GWAS-identified loci (36). The SMR framework combines GWAS and eQTL summary-level data under Mendelian randomization principles to test whether associations between gene expression and a trait arise from a shared causal variant. In this context, cis-eQTL variants were employed as instrumental variables (IVs) for gene expression. Separate SMR analyses were performed for blood and thyroid tissues. For blood-derived expression, eQTL datasets from Westra, CAGE, and GTEx v8 were used (37–39). That the BH method (via R’s p.adjust function) was used for FDR correction. Furthermore, heterogeneity in dependent instruments (HEIDI) testing was applied to assess linkage disequilibrium among the identified associations.

### 2.6 Conditional and joint analysis

Conditional and joint analyses were conducted using the post-processing module of the FUSION pipeline to detect independent TWAS associations within each genomic panel. Genes that met the false discovery rate (FDR) criterion of < 0.05 were included in the conditional analysis. To ensure the robustness of the identified genetic effects, p-values from the TWAS were compared before and after conditioning. Genes that remained statistically significant following conditional adjustment were considered jointly significant.

### 2.7 MR for causal relationship between genes and T1D

Given the relatively small number of SNPs achieving genome-wide significance, we adopted a significance threshold of p < 1 × 10^-5^. Selected variants were required to be spaced at least 10000 kbp apart and exhibit minimal linkage disequilibrium (LD; R² ≤ 0.001). To assess the direction of causality, we applied the MR-Steiger test. A P value < 0.05 was considered supportive of a causal effect from the exposure to the outcome. The strength of instrumental variables (IVs) was assessed using the proportion of variance explained (R²) and the F-statistic (40). R² was derived as 2 × (1 − MAF) × MAF × β², where MAF denotes the minor allele frequency and β represents the effect size of the exposure. The F-statistic was calculated by F = R² × (N − K − 1)/[K × (1 − R²)], with values exceeding 10 indicating a sufficiently strong instrument–exposure association (41). Variants with F-statistics below this threshold were excluded. MR analyses were conducted using at least two SNPs as instruments and implemented through multiple complementary methods, including inverse variance weighting (IVW), weighted median, MR-Egger regression, and MR-Pleiotropy RESidual Sum and Outlier (MR-PRESSO). The IVW approach served as the primary estimator of causal effects (42), whereas the weighted median and MR-Egger models were applied to reduce bias from horizontal pleiotropy, thereby strengthening the robustness of IVW findings (43). MR-PRESSO was further used to detect outlier SNPs potentially influenced by pleiotropic effects. To evaluate the statistical power of significant causal associations, we used an online tool (https://shiny.cnsgenomics.com/mRnd/). Heterogeneity among selected instruments was examined using Cochran’s Q test (p < 0.05); when significant heterogeneity was detected, a random-effects IVW model was applied for more conservative estimation. The MR-Egger intercept was used to test for directional pleiotropy. Finally, scatter plots were generated to illustrate causal effect estimates for individual variants, and “leave-one-out” sensitivity analyses—visualized through forest plots—were conducted to assess the stability of the overall results. For several genes, only one or two independent cis-eQTLs were available as instrumental variables. While this reflects the biological architecture of gene regulation, analyses with few IVs have reduced statistical power and are more sensitive to potential biases from a single variant. To mitigate these concerns, we rigorously assessed instrument strength (F-statistic > 10), performed leave-one-out sensitivity analyses, and employed robust MR methods (weighted median estimator).

### 2.8 T1D single-cell RNA sequencing-based analyses

To examine the potential mediation of peripheral immune cell activity at the single-cell level, we analyzed transcriptomic data from 117,737 immune cells derived from 77 samples, including 31 from healthy controls and 46 from patients (44). Quality-control criteria were applied as follows: minGene = 200, maxGene = 3,000, and pctMT = 5%. A total of 5,000 highly variable genes were retained for downstream analyses, and batch effects were corrected using the Harmony package in R. We performed integration using Harmony with all default parameters (i.e., nclust = 50, max_iter = 10). This was because preliminary testing showed that the default settings were sufficient to effectively remove the major batch effects in our data while preserving the expected biological cluster structure. Following clustering, cell populations were manually annotated according to canonical marker expression: NK cells(NCR1), CD4+T cells(IL7R), CD8+T cells(CD8B), T-reg(FOXP3), B_Naive(IGHD), B_SM(CR2), B_plasma(IGHG1), MAIT cells(SLC4A10), I_Monocytes(FCGR3A,CD14), C_Monocytes(CD14), NC_Monocytes(FCGR3A), Platelets(PPBP), HSCs(CD34), Erythrocytes(HBB), cDCs(CD1C), and pDCs(IRF7). Marker genes used for annotation were obtained from the CellMarker database and previous studies (45–47).

### 2.9 T1D single-cell RNA joint analysis with GWAS

Cell type enrichment within the type 1 diabetes (T1D) single-cell RNA-seq datasets was evaluated using scDRS (version 1.0.3) (48). Gene-level P-values and Z-scores were first derived from T1D GWAS summary statistics with MAGMA (version 1.10) (29), and the top 2,000 ranked genes were selected as candidate disease-associated genes. scDRS then computed disease scores for each cell in the single-cell datasets, generating Monte Carlo control scores based on random gene sets. The resulting scores were normalized, and P-values were obtained for individual cells. Associations between disease-related gene sets and specific cell populations were estimated using the compute_downstream function with default parameters. In parallel, scPagwas (version 1.1.0) (49) was applied to calculate trait-relevant scores (TRS) for individual cells, based on the top 1,000 trait-associated genes using Seurat’s AddModuleScore function (50). scPagwas assessed statistical significance through percentile-rank testing and identified T1D-associated predefined cell types via a block-bootstrap approach (30). Only autosomal SNPs with a minor allele frequency (MAF) > 0.01 were included, while variants within the major histocompatibility complex (MHC) region (chromosome 6: 25–35 Mbp) were excluded owing to extensive linkage disequilibrium (LD) (51).

### 2.10 Gene set enrichment analysis

Patients were stratified into high- and low-expression groups based on gene expression levels. Gene set enrichment analysis (GSEA) was performed to identify differences in signaling pathway activity between the two groups (52). Annotated pathway gene sets were obtained from the Molecular Signatures Database (MSigDB). Pathway-level differences were assessed by enrichment analysis, and significantly enriched gene sets were identified using consistency scores with an adjusted P value < 0.05.

### 2.11 Gene–compound interaction analysis and molecular docking

Gene–drug interaction analysis was performed to identify potential therapeutic candidates for T1D using the Drug–Gene Interaction (DGI) database. The DGI resource integrates curated information on druggable genes and gene–drug interactions from multiple published studies, public repositories, and web-based platforms (53). Compound exhibiting an interaction score greater than zero with jointly significant genes were considered potential therapeutic agents for T1D. For molecular docking analysis, The molecular structures of candidate Compounds were downloaded from PubChem or ChEMBL in SDF format (54). Docking simulations were performed using AutoDock Vina (55), with the following key parameters: the grid box size was set to 30 × 30 × 30 Å centered on the predicted or canonical binding pocket, and the exhaustiveness parameter was set to eight. Docking site selection was based on either the known ligand binding pocket or the most probable surface cavity predicted by AutoSite. Compounds with binding free energy ≤ −6 kcal/mol were considered to have high affinity and potential therapeutic relevance.

### 2.12 Phenome-wide association analysis

To further assess potential horizontal pleiotropy of candidate drug targets and evaluate possible adverse effects, a phenome-wide association study (PheWAS) was conducted using the AstraZeneca PheWAS Portal (https://azphewas.com/). The original PheWAS dataset included approximately 15,500 binary and 1,500 continuous phenotypes derived from exome sequencing data of roughly 450,000 participants in the UK Biobank. Detailed information on the construction and design of this dataset is available in the primary publication (56). Multiple-testing correction was applied, with a significance threshold of p < 2 × 10^-9^, consistent with the default setting of the AstraZeneca PheWAS Portal, to minimize the likelihood of false-positive findings.

### 2.13 Statistical analysis

All statistical analyses were conducted using Ubuntu 22/Bash (GNU Project Bourne Again Shell) and R version 4.2.1 (The R Project for Statistical Computing, Vienna, Austria).

## 3 Results

### 3.1 Tissue type enrichment analysis

**Figure 2:**
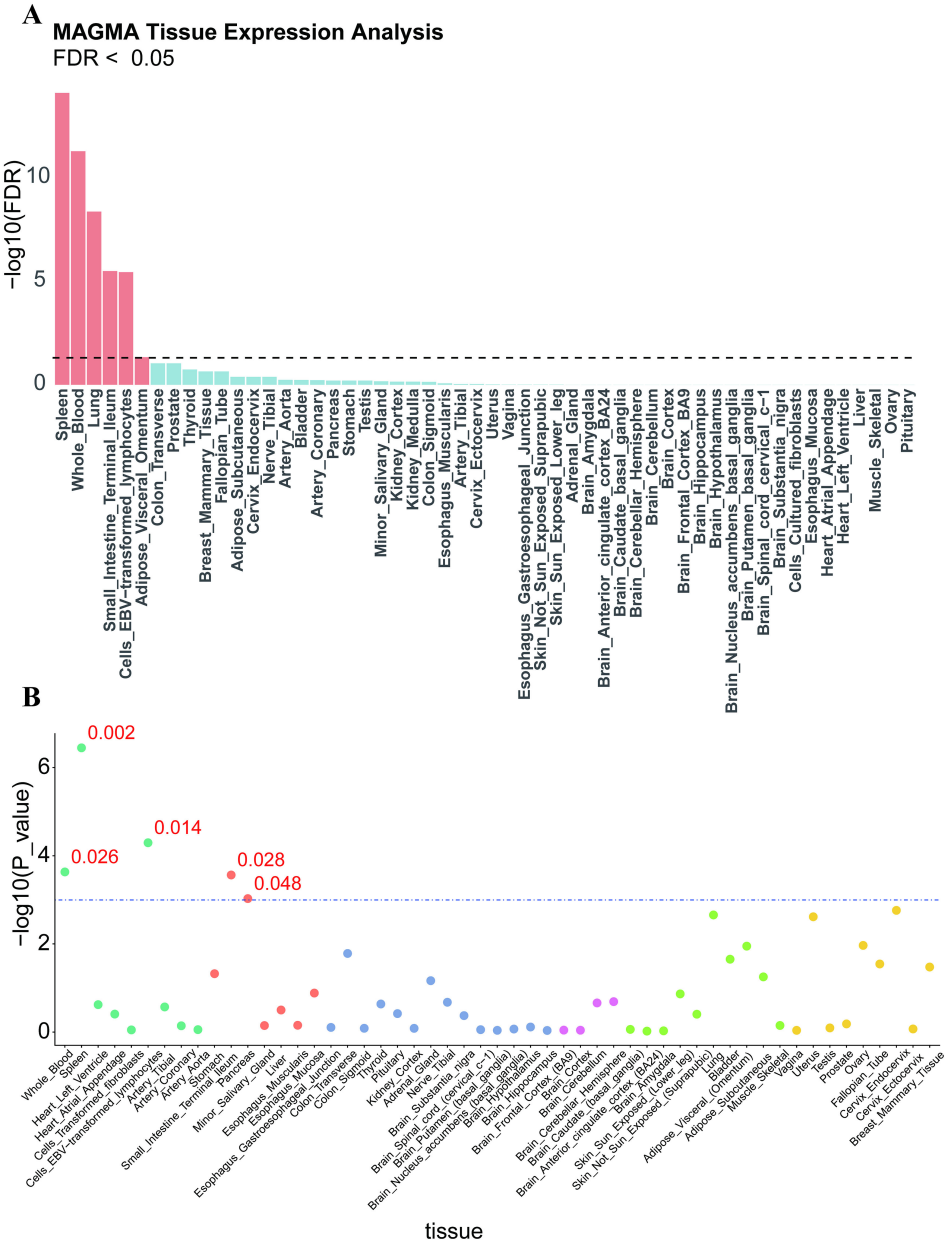
**(A)** MAGMA method results for tissue enrichment of T1D in GTEx 8, where red represents significant tissues and blue indicates tissues that did not meet the threshold. The x-axis represents -log(FDR value), and the black dashed line indicates the cutoff value (FDR < 0.05). **(B)** Determination of tissue priority using LDSC-SEG. A bubble chart describing tissue priority as determined by LDSC-SEG. GTEx data is categorized into six major classes. The x-axis represents -log(p-value), and the blue dashed line indicates the cutoff value (p-val < 0.05).

To explore the specificity of tissue-disease relationships, we performed MAGMA and LDSC-SEG analyses. MAGMA, based on gene analysis, identified 830 genes associated with T1D. We also conducted gene enrichment analysis on the 830 T1D-identified genes using GTEx V8. In GTEx V8, our analysis indicated that genes differentially expressed under these conditions were mainly enriched in a series of tissues, including Spleen (Pfdr = 8.1243e-15), Whole_Blood (Pfdr = 5.30712e-12), Lung (Pfdr = 4.38768e-09), Small_Intestine (Pfdr=3.25512e-06),Cells_EBV-transformed_lymphocytes (Pfdr = 3.666492e-06), and Adipose_Visceral_Omentum (Pfdr = 0.0463149) (**Figure 2A**). In the LDSC-SEG analysis, Spleen, Cells_EBV-transformed_lymphocytes, Whole_Blood, and Small_Intestine were confirmed to be significantly related to the disease, with the Pancreas also being related to the disease (**Figure 2B**).

### 3.2 Multi-tissue TWAS combined with SMR discovers novel T1D loci

**Figure 3:**
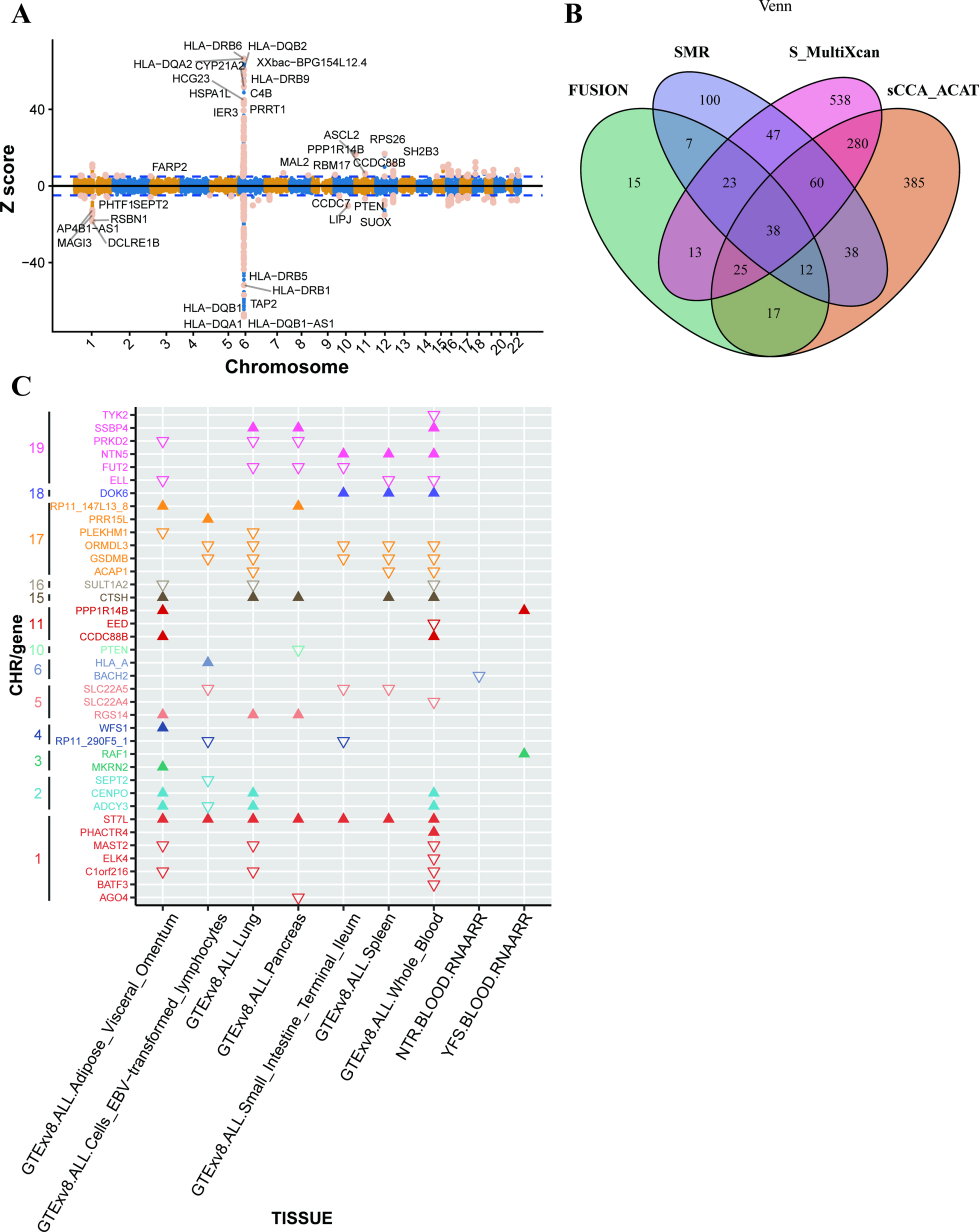
**(A)** Manhattan plot of genes associated through multi-tissue TWAS. Each data point represents a gene, with the x-axis indicating the chromosome number where the gene is located, and the y-axis representing the TWAS-Z score of the TWAS signal. Genes that pass the threshold (Bonferroni-corrected P < 0.05) are highlighted in orange. If a gene is identified in multiple tissues, it is marked with the highest absolute value. **(B)** Integrated results from TWAS, SMR, S-MultiXcan, and sCCA-ACAT analyses. **(C)** Distribution of z-scores in significant gene-tissue pairs. Genes are grouped by chromosome (y-axis) and their respective tissues (x-axis). Upward and downward triangles represent positive and negative z-scores, respectively.

We employed FUSION for TWAS to identify susceptibility genes for T1D. Utilizing the six tissue-specific analyses from MAGMA that showed significant False Discovery Rate (FDR), namely spleen, whole blood, lung, small intestine, EBV cells, and visceral fat, along with the pancreas which is closely related to T1D, we analyzed all genes involved in seven tissues from the GTEx v8 data, as well as whole blood and peripheral blood data from the NTR and YFS studies. Out of a total of 56,141 associations, 957 genes at 327 loci were significantly associated (Bonferroni-corrected P-value < 0.05) (**Figure 3A**). Four hundred and thirteen blood tissue-specific genes from the three datasets were significantly detected by FUSION with an FDR less than 0.05. After collocation analysis, 53 genes were obtained (PPH4 > 0.8). The other six tissue datasets from GTEx v8, after significant analysis by FUSION and collocation analysis, yielded the following results: Spleen: 37, Lung: 50, Small Intestine: 34, EBV: 33, Pancreas: 36, Visceral Fat: 46. All significant genes from the FUSION analysis are displayed in (**Supplementary Table S1**).

To enhance the power compared to single-tissue analysis, we utilized S-MultiXcan to merge results from different single-tissue models into a single aggregated statistic. After correction for multiple testing, we identified 1,025 genes significantly associated across all 49 GTEx tissues (multi-tissue FDR < 0.05) (**Supplementary Table S3**).To improve the accuracy of the cross-tissue analysis, we conducted sCCA-ACAT, applying the ACAT method to the sCCA data across 49 tissues, using sparse canonical correlation analysis to enhance the capability of transcriptome-wide association studies. We ultimately identified 856 genes significantly associated across all 49 GTEx tissues (multi-tissue FDR < 0.05) (**Supplementary Table S4**).

To further confirm the tissue-specific genes associated with various tissues, we performed SMR analysis on the eQTL data of the seven tissues (blood data from CAGE.sparse, westra_eqtl_hg19, and GTEx, with the rest from GTEx). Using the CAGE, Westra, and GTEx blood eQTL summary data, we identified 219 probes that were significantly associated with T1D. Utilizing the T1D GWAS summary data, we discovered several probes marking CTSH that demonstrated strong pleiotropy with T1D (β [SE] = 0.181091 [0.021569], PSMR = 4.623454e-17, P HEIDI = 0.07052955, in the CAGE eQTL study; β [SE] = 0.217065 [0.0252317], PSMR = 7.774635e-18, P HEIDI = 0.07546363, in the Westra eQTL study; β [SE] = 0.333205 [0.0435052], PSMR = 1.874229e-14, P HEIDI = 0.3976903, in the GTEx eQTL study). The significant number of genes obtained from the SMR analysis of the other six tissues in GTEx, after applying an FDR less than 0.05 and HEIDI greater than 0.05, are as follows: Spleen: 55, Lung: 65, Small Intestine: 23, EBV: 32, Pancreas: 47, Visceral Fat: 65. (**Supplementary Table S2**) This analysis strengthens the evidence for the role of these genes in T1D and provides a basis for further exploration of their biological functions and potential as therapeutic targets. The use of SMR analysis across multiple tissues helps to elucidate the complex genetic architecture of T1D and the diverse physiological processes that may be implicated in its pathogenesis.

Taking the intersection of genes obtained from the above four methods (**Figure 3B**), a total of 38 genes were obtained. In the 9 tissue panels, there were 90 jointly significant associations (**Figure 3C**), including the same gene loci being associated with the disease in multiple tissues.

### 3.3 Conditional and joint analysis combined with MR on the association between susceptibility genes and T1D

**Figure 4:**
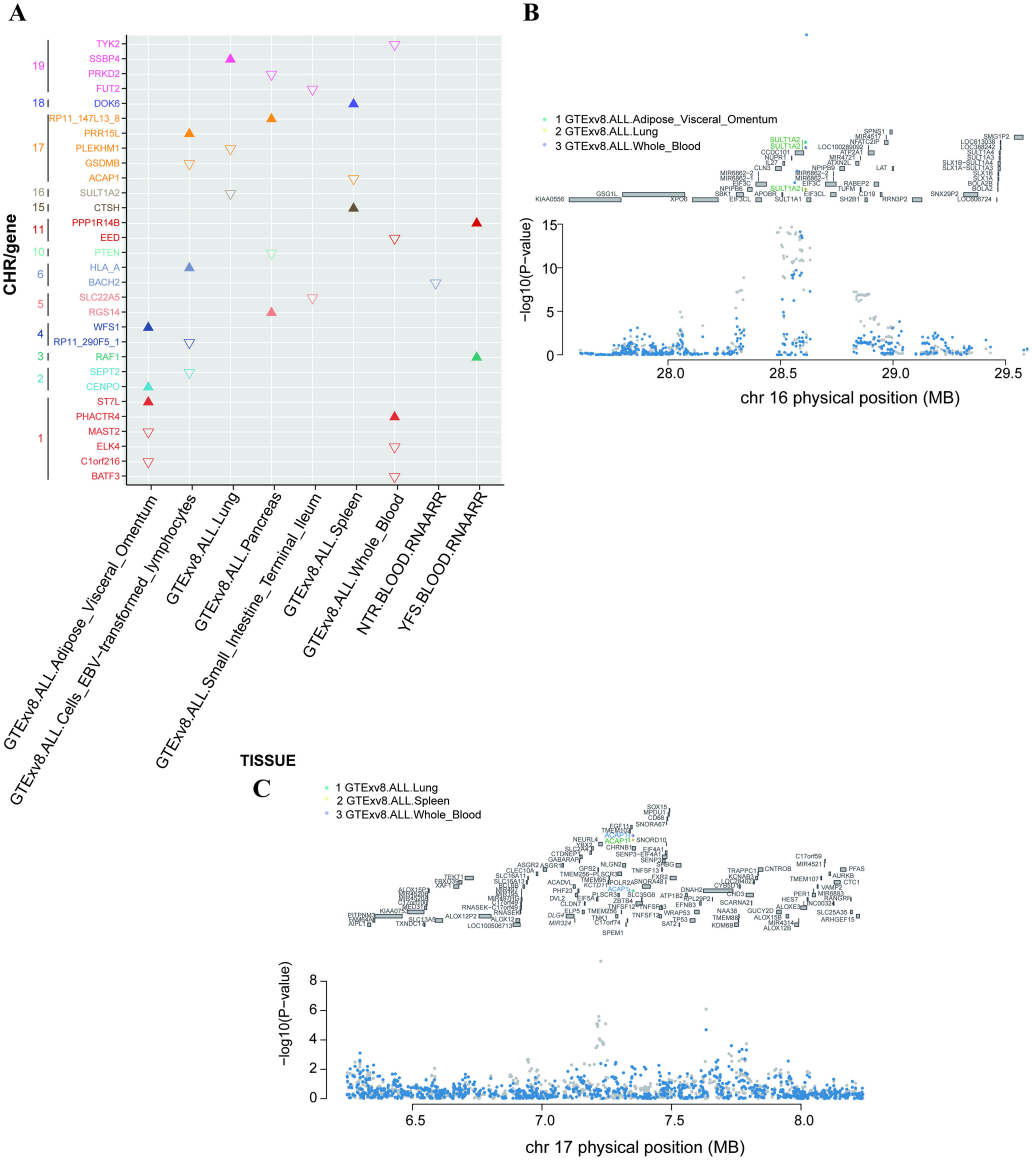
**(A)** Distribution of z-scores in 30 significant gene-tissue pairs among the 33 jointly significant associations. Genes are grouped by chromosome (y-axis) and their respective tissues (x-axis). Upward and downward triangles represent positive and negative z-scores, respectively. **(B)** Results of the conditional and joint analysis for the SULT1A2 gene. Regional association plot on chromosome 16. The green gene at the top of the figure represents the jointly significant gene that best explains the GWAS signal. Colored dots next to the gene indicate the tissue panels that identified the gene. The gray bar indicates the gene’s location on chromosome 16. The lower plot shows the Manhattan plot of GWAS signals. Gray and blue dots represent GWAS-p values before (gray) and after (blue) pre-processing of the jointly significant gene. **(C)** Results of the conditional and joint analysis for the ACAP1 gene. Regional association plot on chromosome 17. The green gene at the top of the figure represents the jointly significant gene that best explains the GWAS signal, with peripheral TWAS-associated genes highlighted in blue. Colored dots next to the gene indicate the tissue panels that identified the gene. The gray bar indicates the gene’s location on chromosome 17. The lower plot shows the Manhattan plot of GWAS signals. Gray and blue dots represent GWAS-p values before (gray) and after (blue) pre-processing of the jointly significant gene.

To rigorously assess the importance of potential inflation of TWAS signals due to linkage disequilibrium contamination, we performed conditional and joint analyses on all TWAS significant loci across the seven tissues. Since the significance of genes is more important than the direction of regulation, all 90 associated joint significances were tested in subsequent analyses. After removing the expected gene expression, 33 of the 90 joint significant associations still held common significance (**Figure 4A**). Taking SULT1A2 as an example, it was previously identified in three TWAS tissue panels (Lung, Whole_Blood, and Adipose_Visceral_Omentum), and the same three tissues remained statistically significant after conditional and joint analyses (**Figure 4B**). At the same time, for ACAP1, which was previously identified in three TWAS tissue panels (Lung, Spleen, and Whole_Blood), only the Spleen tissue remained significant. Additionally, after adjustment in all tissues, the number of genes in each tissue relatively decreased (**Figure 4C**).

Among the 33 joint significant associations, 30 genes were identified, which are represented below as T1D characteristics, including 2 non-coding RNAs and 28 protein-coding genes (**Table 1**).

**Table 1:** Tissue-specific results of novel genes from conditional and joint analysis.

ID	TWAS.Z	TWAS.P	JOINT.BETA	JOINT.BETA.SE	JOINT.Z	JOINT.P	Tissue
BATF3	-5.6	2.80E-08	-5.6	1	-5.6	2.80E-08	Whole_Blood
ELK4	-5.3	1.20E-07	-5.3	1	-5.3	1.20E-07	Whole_Blood
PHACTR4	4.1	4.00E-05	4.1	1	4.1	4.00E-05	Whole_Blood
MAST2	-5.2	2.00E-07	-5.2	1	-5.2	2.00E-07	Adipose_Visceral_Omentum
ST7L	4.8	1.60E-06	4.8	1	4.8	1.60E-06	Adipose_Visceral_Omentum
C1orf216	-4.6	4.50E-06	-4.6	1	-4.6	4.50E-06	Adipose_Visceral_Omentum
PTEN	-10	5.10E-24	-10	1	-10	5.10E-24	Pancreas
EED	-4.2	2.70E-05	-4.2	1	-4.2	2.70E-05	Whole_Blood
PPP1R14B	6.6	4.70E-11	6.6	1	6.6	4.70E-11	YFS.BLOOD.RNAARR
CTSH	10	1.70E-23	10	1	10	1.70E-23	Spleen
SULT1A2	-9.2	2.30E-20	-25	3.2	-7.9	2.00E-15	Lung
PRR15L	6	2.20E-09	6	1	6	2.20E-09	Cells_EBV-transformed_lymphocytes
GSDMB	-5.2	1.60E-07	-5.2	1	-5.2	1.60E-07	Cells_EBV-transformed_lymphocytes
PLEKHM1	-6.3	2.40E-10	-6.3	1	-6.3	2.40E-10	Lung
ACAP1	-6.2	4.30E-10	-6.2	1	-6.2	4.30E-10	Spleen
RP11-147L13.8	4.2	2.30E-05	4.2	1	4.2	2.30E-05	Pancreas
DOK6	7.3	2.90E-13	7.3	1	7.3	2.90E-13	Spleen
SSBP4	4.9	9.80E-07	4.9	1	4.9	9.80E-07	Lung
FUT2	-8.7	3.30E-18	-8.7	1	-8.7	3.30E-18	Small_Intestine_Terminal_Ileum
PRKD2	-8.6	8.90E-18	-8.6	1	-8.6	8.90E-18	Pancreas
TYK2	-5.6	2.00E-08	-5.6	1	-5.6	2.00E-08	Whole_Blood
SEPT2	-5.8	7.10E-09	-5.8	1	-5.8	7.10E-09	Cells_EBV-transformed_lymphocytes
CENPO	5.2	2.20E-07	5.2	1	5.2	2.20E-07	Adipose_Visceral_Omentum
RAF1	4.7	2.80E-06	4.7	1	4.7	2.80E-06	YFS.BLOOD.RNAARR
RP11-290F5.1	-5	7.20E-07	-3.6	1.1	-3.1	0.0016	Cells_EBV-transformed_lymphocytes
WFS1	4.4	9.40E-06	4.4	1	4.4	9.40E-06	Adipose_Visceral_Omentum
SLC22A5	-5.2	1.90E-07	-5.2	1	-5.2	1.90E-07	Small_Intestine_Terminal_Ileum
RGS14	4.5	5.90E-06	4.5	1	4.5	5.90E-06	Pancreas
HLA-A	9	1.80E-19	9	1	9	1.80E-19	Cells_EBV-transformed_lymphocytes
BACH2	-10	2.60E-24	-10	1	-10	2.60E-24	NTR.BLOOD.RNAARR

Taking the aforementioned 28 genes as exposures and Type 1 Diabetes (T1D) as the outcome, we extracted exposure SNPs based on linkage disequilibrium with parameters p < 1e-5, kb = 10000, and r2 = 0.001 in various tissue panels for different genes, followed by MR analysis with the outcome. Among the 28 genes, many lacked SNPs due to linkage disequilibrium values, and some showed no causal relationship as the inverse-variance weighted (IVW) method yielded p-values > 0.05. Ultimately, the MR analysis supported a putative causal role for 10 genes, including 7 not previously reported in T1D contexts (**Table 2**). The F-statistics for all instrument variables exceeded 10 (**Supplementary Table S5**). No statistically significant horizontal pleiotropy was detected, supporting the validity of the primary inverse-variance weighted analyses. Despite heterogeneity, the pleiotropy-robust weighted median estimates showed consistent direction and comparable magnitude (**Supplementary Table S6**). Furthermore, leave-one-out sensitivity analysis did not identify any single SNP as the primary driver of heterogeneity (**Supplementary Figure S1**). Furthermore, the MR-Steiger directionality test confirmed that the causal direction for all significant associations was from gene expression to T1D risk (Steiger P < 0.05; **Supplementary Table S6**), effectively ruling out reverse causation. There is a significant association between higher genetically determined gene expression of PHACTR4, ST7L, and WFS1 and an increased risk of T1D. Conversely, lower gene expression of ELK4, MAST2, C1orf216, and SULT1A2 is associated with an increased risk of T1D.

**Table 2:** Mediation Mendelian randomization analyses of the causal effects between 10 genes and T1D.

ID	MR.nsnp	MR.b	MR.se	MR.pval	JOINT.Z	Tissue	Reported
BATF3	17	0.258187824	0.054644266	2.30E-06	-5.6	Whole_Blood	yes
ELK4	7	0.592258602	0.292121675	0.042617633	-5.3	Whole_Blood	no
PHACTR4	45	-0.107582285	0.022295081	1.40E-06	4.1	Whole_Blood	no
MAST2	2	0.36624904	0.068953845	1.09E-07	-5.2	Adipose_Visceral_Omentum	no
ST7L	7	-0.09173561	0.036021513	0.010875014	4.8	Adipose_Visceral_Omentum	no
C1orf216	4	0.237367931	0.060286947	8.24E-05	-4.6	Adipose_Visceral_Omentum	no
SULT1A2	1	-0.087881447	0.02131953	3.75E-05	-7.9	Whole_Blood	no
CENPO	3	-0.229140648	0.035132082	6.93E-11	5.2	Adipose_Visceral_Omentum	yes
WFS1	1	-0.233162406	0.057052656	4.37E-05	4.4	Adipose_Visceral_Omentum	no
HLA-A	7	-0.29847205	0.058469389	3.31E-07	9	Cells_EBV-transformed_lymphocytes	yes

### 3.4 Cell type and gene set enrichment analysis

**Figure 5:**
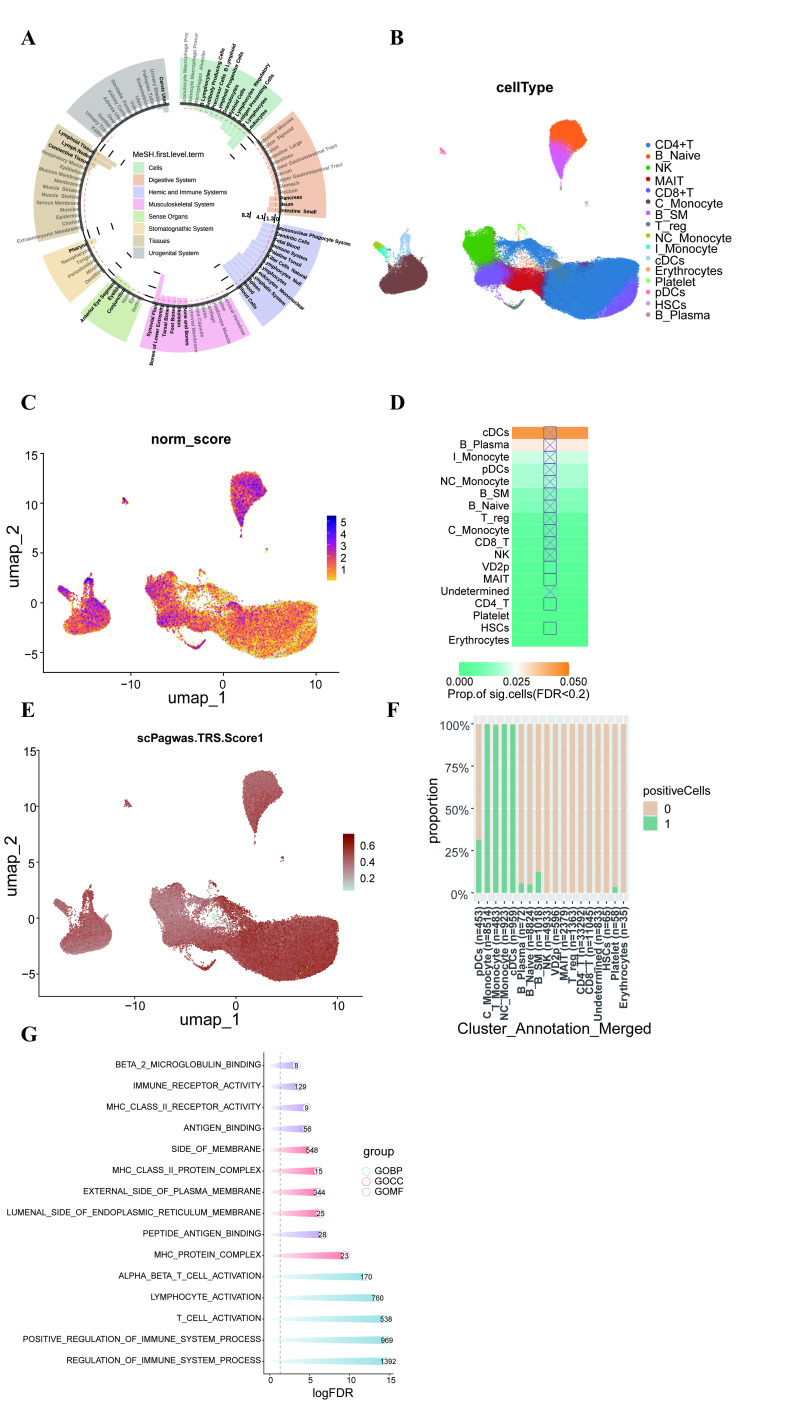
**(A)** Tissue and cell type enrichment analysis performed using DEPICT. The y-axis represents -log10(P-value). The x-axis displays the first-level Medical Subject Headings (MeSH) annotations. A strong enrichment for blood and immune cells is observed. The red horizontal line corresponds to -log10(0.05) = 1.3. **(B)** Single-cell UMAP (Uniform Manifold Approximation and Projection) plot. **(C)** UMAP (Uniform Manifold Approximation and Projection) plot displaying the scDRS (single-cell Disease Risk Score) scores for disease phenotypes. **(D)** Heatmap colors indicate the proportion of cells significantly associated with each cell type trait; squares represent significant associations (FDR < 0.1) between cell types and traits across all cell type and trait combinations; cross symbols indicate significant heterogeneity in traits associated with individual cells within a specific cell type. **(E)** UMAP plot showing the scPagwas (single-cell Page’s test) TRS (Transcriptional Regulatory Score) scores for disease phenotypes. **(F)** Proportion of positive cells (cells with TRS scores adjusted P-value less than 0.05) in each cell subpopulation. **(G)** Results of the GO analysis, categorized into BP (Biological Process), CC (Cellular Component), and MF (Molecular Function). The numbers inside the circles represent the count of genes involved in each pathway. The x-axis represents -log(FDR), and the gray dashed line indicates the cutoff value (FDR < 0.05).

To understand the cell and tissue types associated with T1D, we conducted DEPICT analysis. Based on the results of DEPICT analysis (**Figure 5A**), in the analysis related to physiological systems, T1D showed the most significant association with the hematopoietic immune system. In the analysis related to cell types, there was a correlation with blood cells, antibody production, and antigen presentation. In the analysis related to tissue types, a significant association with lymphoid tissue was observed. In addition to these analyses, we aimed to identify T1D-related cell subpopulations by combining GWAS summary statistics with human peripheral blood scRNA-seq data using the scDRS method. After quality control and data filtering, we obtained single-cell transcriptomes of 117,737 immune cells from 77 samples (31 healthy, 46 diseased). To identify the main populations and subpopulations of peripheral blood immune cells in T1D, we used Seurat for clustering and identified 16 immune cell types, including NK cells(NCR1), CD4+T cells(IL7R), CD8+T cells(CD8B), T-reg(FOXP3), B_Naive(IGHD), B_SM(CR2), B_plasma(IGHG1), MAIT cells(SLC4A10), I_Monocytes(FCGR3A,CD14), C_Monocytes(CD14), NC_Monocytes(FCGR3A), Platelets(PPBP), HSCs(CD34), Erythrocytes(HBB), cDCs(CD1C), and pDCs(IRF7) (**Figure 5B**). FeaturePlot results confirmed the accuracy of the cell type annotation (**Supplementary Figure S2**). Among the 16 major cell types analyzed, we found significant enrichment of several key immune populations in T1D, with conventional dendritic cells (cDCs) showing the highest enrichment score (**Figures 5C, D**). This aligns with their central role as professional antigen-presenting cells capable of activating autoreactive T cells. Furthermore, applying the cell-scoring method scPagwas to the same data revealed significantly higher trait-related scores (TRS) in cDCs, NC monocytes, C monocytes, I monocytes (**Figure 5E**), cell types implicated in inflammatory cytokine production and antigen presentation. Within these subpopulations, a significantly higher proportion of cells was classified as TRS-positive (TRS-adjusted p-value < 0.05) (**Figure 5F**), collectively underscoring the pronounced involvement of innate immune antigen-presenting cells in T1D pathology.

Furthermore, a Gene Ontology(GO) analysis was specifically conducted on the 830 T1D-associated genes identified by MAGMA. The results showed that biological processes (BP) related to the regulation of immune system processes and the activation and differentiation of T cells were enriched. In terms of cellular components (CC), these genes showed enrichment in MHC protein complexes. In terms of molecular functions (MF), genes were enriched for functions such as peptide-antigen binding. The most representative GO terms are shown in the figure. The enrichment analysis indicates that these genes play a key role in the regulation of cytokines, biosynthetic processes, and cytokine-mediated signaling pathways, which also supports the close relationship between T1D and immune system disorders (**Figure 5G**).

### 3.5 Gene localization at single-cell level and single-gene enrichment analysis

**Figure 6:**
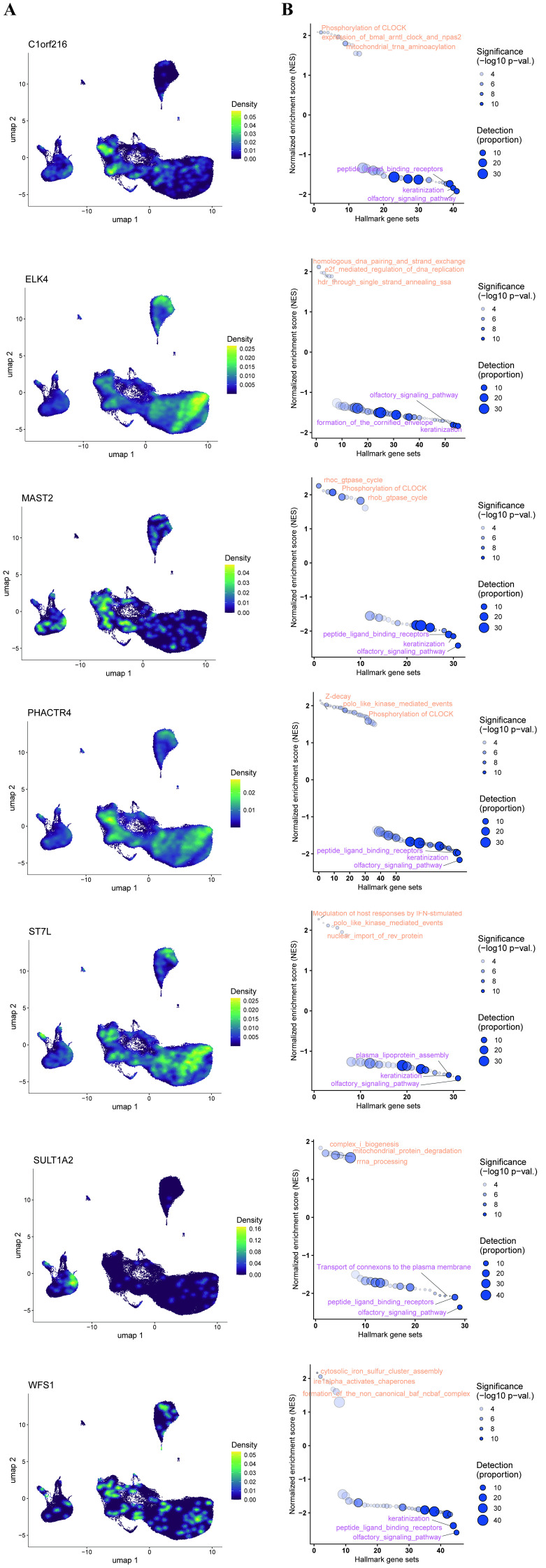
**(A)** UMAP visualization showing the cell type-specific expression patterns of seven key genes (C1orf216, ELK4, MAST2, PHACTR4, ST7L, SULT1A2, and WFS1) across major immune cell subsets. **(B)** Single-gene Gene Set Enrichment Analysis (GSEA) for each gene, depicting enriched biological pathways and their normalized enrichment scores (NES). Key associated pathways are labeled.

Single-cell transcriptomic analysis revealed distinct cell type-specific expression patterns for multiple genes, along with their potential functional associations as indicated by single-gene Gene Set Enrichment Analysis (GSEA) (**Figure 6**). Specifically, C1orf216 and ELK4 were specifically highly expressed in cytotoxic immune cells (CD8^+^ T and NK cells) and CD4^+^ T cells, respectively, potentially involved in the phosphorylation of CLOCK protein and homologous DNA pairing and strand exchange. MAST2 and PHACTR4 showed predominant expression in innate immune cells. MAST2 was broadly expressed in CD8^+^ T cells, NK cells, and classical monocytes (C_MONO) and may be associated with the Rhoc GTPase cycle, whereas PHACTR4 was specifically highly expressed in NK cells and might participate in the Z-decay pathway. ST7L and SULT1A2 were primarily enriched in CD4^+^ T cells/non-classical monocytes (NC_MONO) and C_MONO, respectively, with potential functional roles in the modulation of host responses by IFN-stimulated genes and mitochondrial complex I biogenesis. Notably, WFS1 expression showed no distinct cell type specificity, and its function may be related to cytosolic iron-sulfur cluster assembly.

### 3.6 Potential candidate compounds for T1D

To prioritize testable hypotheses for drug repurposing or development, we performed a gene-compound interaction analysis as an initial computational screen. Using the seven newly identified susceptibility genes as queries, the Drug-Gene Interaction Database (DGIdb) was interrogated. This preliminary in silico analysis identified putative interactions for only three of the seven genes, yielding six unique gene-compound pairs with interaction scores above zero (two of which are previously validated). The interaction between COMPOUND 5G and ELK4 received the highest database-derived score (**Table 3**).

**Table 3:** Drug enrichment analysis of susceptibility genes.

Gene	Drug	Regulatory approval	Interaction score
WFS1	CISPLATIN	Approved	0.7102557627059256
ELK4	DCLK1-IN-1	Not Approved	6.525474819860691
ELK4	COMPOUND 5G	Not Approved	13.05094963972138
SULT1A2	ENDOXIFEN	Not Approved	11.60084412419678
SULT1A2	4-HYDROXYTAMOXIFEN	Not Approved	6.960506474518071
SULT1A2	STREPTOZOCIN	Approved	0.756576790708486

### 3.7 PheWAS

**Figure 7:**
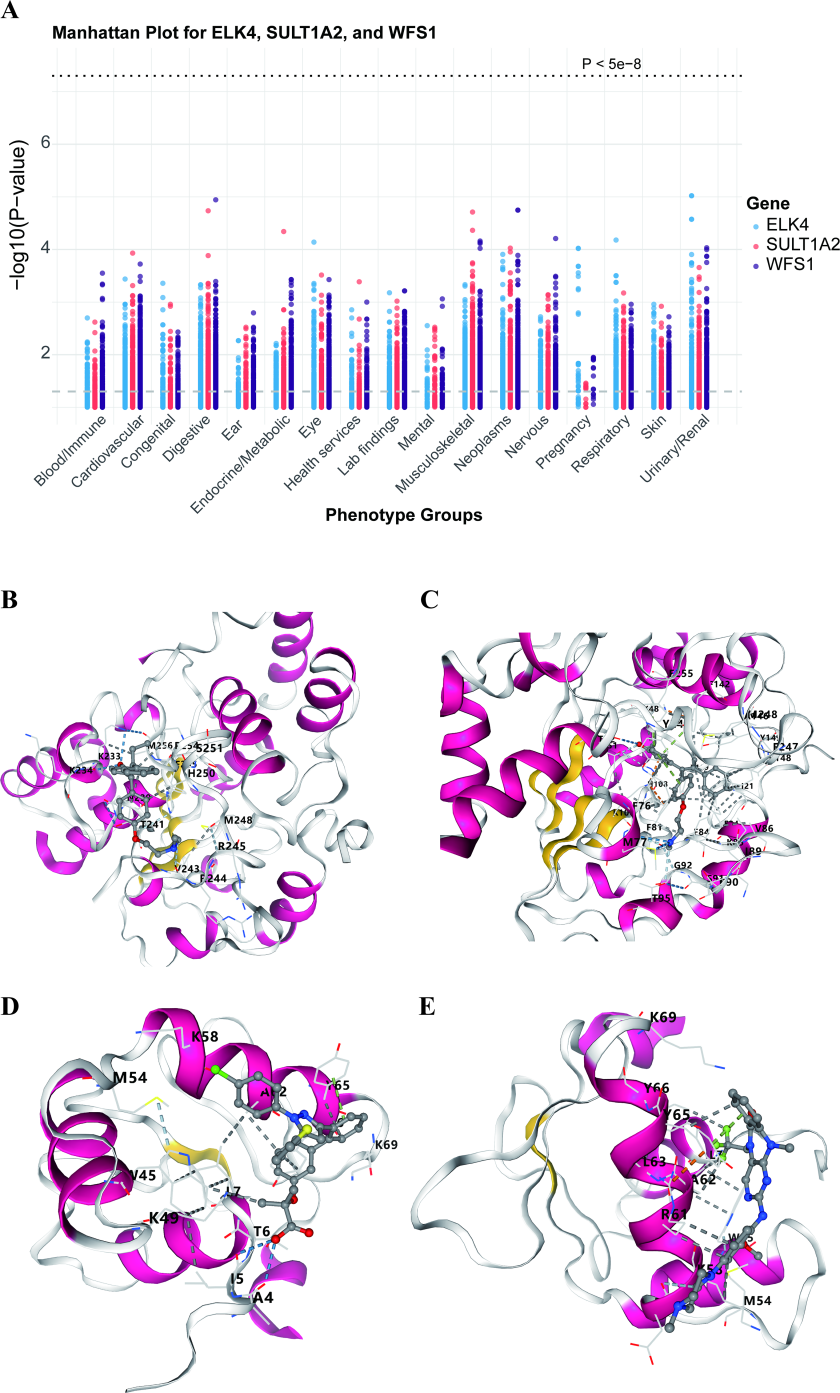
**(A)** Manhattan plot for phenome-wide MR results of SULT1A2, ELK4 and WFS1. Ordinate representation of the p value in phenome-wide MR results. A dot represents a disease trait, and different colors represent the MR result of different expressions. **(B-E)** docking models of the best combinations (from top left to bottom right: SULT1A2 and 4-Hydroxytamoxifen, SULT1A2 and Endoxifen, ELK4 and COMPOUND5G, and ELK4 and Dclk1-IN-1).

To further assess whether the three identified potential drug targets might have beneficial or adverse effects on other traits, as well as to evaluate potential pleiotropy not captured by the MR-Egger intercept test, this study utilized 17,361 binary phenotypes and 1,419 quantitative phenotypes from the AstraZeneca PheWAS Portal database to perform PheWAS at the gene level. PheWAS results can be interpreted as associations between genetically determined protein expression and specific diseases or traits. None of the three drug targets were significantly associated with other traits at the gene level (genome-wide association P < 5E-8) (**Figure 7A**). This suggests that drugs targeting these genes may have minimal potential side effects and pleiotropy at the gene level, further supporting the robustness of the study findings.

### 3.8 Molecular docking

To evaluate the potential affinity of the computationally prioritized compounds for their predicted targets, we performed molecular docking analysis using Autodock Vina v.1.2.2. The binding poses and interactions of the four top-predicted compounds with the protein structures of their corresponding target genes were analyzed. Calculated binding energies suggested favorable in silico binding for all four compound-protein pairs (**Table 4**, **Figures 7B–E**). The docking models indicated the formation of hydrogen bonds and electrostatic interactions in each case. Notably, the docking pose for ELK4 and DCLK1-IN-1 showed the lowest calculated binding energy (-7.4 kcal/mol) among the pairs analyzed. These computational results provide initial structural hypotheses for target engagement that require experimental validation.

**Table 4:** The binding energy of protein-small molecule docking.

Target	Drug	Binding energy
ELK4	DCLK1-IN-1	-7.4
ELK4	COMPOUND 5G	-7.2
SULT1A2	4-HYDROXYTAMOXIFEN	-7
SULT1A2	ENDOXIFEN	-7.2

Binding energy: quantifies the interaction strength between the target and drug. More negative values denote stronger binding affinity, while values near zero indicate weaker binding.

## 4 Discussion

T1D is a complex autoimmune disease involving various genetic and environmental factors in its pathogenesis. Traditionally, research has mainly focused on known susceptibility genes, such as those in the HLA region (57), but these genes only account for part of the genetic risk. In this study, we utilized multi-tissue TWAS and mendelian randomization analysis to identify 10 T1D-associated genes, 7 of which have not been previously reported. The discovery of these genes significantly expands our understanding of the genetic basis of T1D. We also explored the immune cell subsets associated with T1D by integrating GWAS data with single-cell data. The study found that specific immune cell subsets, such as cDC, pDC, B cells, and CD8+ effector T cells, were significantly enriched in T1D. These cell subsets may play crucial roles in the pathogenesis of T1D, offering new perspectives for future therapeutic strategies. Three druggable genes significantly associated with T1D were identified through DGIdb, along with six drug-gene pairs. To further elucidate the potential pleiotropy and drug side effects of these key druggable genes, phenome-wide association studies were conducted on three SNPs related to the genes of interest (SULT1A2, WFS1, and ELK4). Finally, molecular docking analysis revealed highly stable interactions and significant druggable potential.

One of the strengths of our research methodology lies in the application of TWAS to investigate the genetic influence on the pathogenesis of various diseases. The advantage of TWAS is that it utilizes large-scale GWAS summary statistics to calculate expected gene expression values, thereby estimating genotype-mediated changes in gene expression at the population level (58–61). In the GWAS summary statistics we employed, healthy subjects (n = 501,638) outnumbered T1D patients (n = 18,942) by approximately 26 times, which could potentially bias the results. Therefore, we calculated the effective sample size, and the case-control ratio did not appear to introduce bias in the outcomes. Given that these genes can exhibit different expression patterns across various tissues, it is crucial to select the appropriate tissue for tissue-specific analysis (62). We utilized multi-tissue RNA expression datasets. Based on the prioritized sorting of tissues, we included other relevant tissues not used in previous T1D TWAS studies (spleen, lung, small intestine, EBV cells, and visceral fat) (63). Previous studies have shown that these tissues are associated with T1D (64–71). Some of the marker genes identified in this study may be partly due to the addition of new tissue panels. The use of multi-tissue panels with S-MultiXcan and sCCA-ACAT also considered tissue-specific and tissue-shared effects. When studying the pathogenesis of diseases, determining tissue-specific effects is crucial; however, most genetic effects are shared across many different tissues (72). Notably, the TWAS results using whole blood and spleen showed a high degree of similarity. Therefore, it is necessary to integrate both tissue-specific and tissue-shared effects to identify reliable genetic markers for the disease. We recognize that signals from non-islet tissues may arise indirectly (e.g., through pleiotropy or LD) rather than reflecting direct roles in T1D. To minimize false positives, we applied strict multiple-testing correction and required cross-validation across complementary TWAS methods. Candidate genes then underwent colocalization and SMR-HEIDI analysis. This multi-step pipeline ensures robust identification of high-confidence T1D candidates, even from less conventional tissues.

In addition to utilizing seven different tissue panels highly relevant to T1D, we also performed four distinct gene prioritization methods (TWAS, SMR, S-MultiXcan, and sCCA-ACAT). We believe that integrating these four approaches can complement the shortcomings of each method and we identified 38 important genes for T1D. To eliminate the inflation potentially caused by LD contamination and enhance the causal association with the disease, we conducted conditional joint analysis and medelian randomization analysis, ultimately identifying 10 significant pathogenic genes,7 of which have not been previously reported. These findings provide new perspectives for genetic research in T1D.

ELK4 functions autonomously in the thymus, regulating the generation of innate αβ CD8^+^ T cells with memory-like characteristics. Accordingly, ELK4 may influence the onset and progression of T1D by modulating immune cell activity and function (73). Reduced PHACTR4 expression has been associated with abnormal endothelial proliferation, neovascularization, and inflammatory responses marked by infiltration of activated CD4^+^ and CD8^+^ T cells, B cells, and mast cells, potentially impairing pancreatic β-cell function (74). SULT1A1 and SULT1A2 encode enzymes involved in amine and lipid metabolism and have been implicated in the etiology of both T1D and type 2 diabetes (T2D) through related but distinct metabolic mechanisms (75). Notably, WFS1 differs from the other identified genes in that it is a known monogenic diabetes gene associated with Wolfram syndrome and a common-variant susceptibility locus for T2D. Thus, its association in our analysis likely reflects shared β-cell stress and endoplasmic reticulum dysfunction pathways across diabetes subtypes rather than a T1D-specific genetic signal (76). Consistent with this interpretation, a recent observational study reported that nearly one-quarter of insulin antibody–negative Indian children with T1D harbor recessive mutations in the WFS1 gene (77). Previous GWAS have identified ST7L as a susceptibility gene for T2D (78). Functionally, ST7L inhibits downstream Wnt/β-catenin signaling, disrupts the Th17/Treg balance, and impairs immune tolerance, potentially increasing pancreatic β-cell vulnerability to immune-mediated attack and promoting T1D progression (79). MAST2 regulates lipopolysaccharide-induced NF-op signaling through complex formation with TRAF6, leading to pro-inflammatory cytokine activation and potentially contributing to βontri damage and increased T1D risk (80). Finally, this study is the first to implicate C1orf216 in T1D and to suggest a potential role inxCLOCK protein phosphorylation. Although CLOCK phosphorylation has not previously been linked to T1D pathogenesis, substantial evidence supports an association between circadian rhythm dysregulation, immune homeostasis, and autoimmune disease (81). Given the high expression of C1orf216 in cytotoxic immune cells central to T1D development, we hypothesize that it may modulate immune function through circadian pathways, contributing to the breakdown of self-tolerance. This novel association warrants further experimental validation to elucidate the underlying mechanisms.

We performed gene-compound interaction analysis to generate testable therapeutic hypotheses for T1D. Currently, drugs used or under investigation for T1D treatment include teplizumab, rituximab, and etanercept (82). These drugs target T cells, B cells, and the inflammatory factor IFN-a inhibitors, respectively. Among these, only teplizumab has been approved by the FDA for T1D treatment (83). Based on the six drug-gene pairs obtained from the gene-drug interaction analysis, it is known that COMPOUND 5G inhibits GPR91, a GPCR that plays a crucial role in major metabolic diseases, including metabolic syndrome, diabetes, dyslipidemia, and non-alcoholic fatty liver disease (84). DCLK1-IN-1 is an inhibitor of the DCLK1 gene, and literature suggests that DCLK1-IN-1 significantly alleviates cardiac hypertrophy and fibrosis in STZ-induced diabetic mice (85). Both 4-HYDROXYTAMOXIFEN and ENDOXIFEN are estrogen receptor inhibitors, and studies have shown that estrogen can treat T1D (86), suggesting that 4-HYDROXYTAMOXIFEN and ENDOXIFEN may have toxic effects in T1D. CISPLATIN impairs mitochondrial function and insulin secretion in mouse islets (87). STREPTOZOCIN, as a drug inducing T1D (88), is commonly used to model T1D in mice. In summary, our analysis prioritizes COMPOUND 5G and DCLK1-IN-1, among others, as high-interest hypotheses for future functional validation in T1D models. These predictions, derived from literature-curated associations, must be considered as starting points for experimental drug repurposing studies, which will need to comprehensively address efficacy, specificity, and ADME properties in relevant biological systems. It is critical to emphasize that these predictions, derived from database mining and molecular docking, are strictly hypothesis-generating. Molecular docking provides preliminary insights into potential binding modes but cannot assess key determinants of therapeutic potential, such as binding affinity and specificity under physiological conditions, cellular permeability, metabolic stability, or off-target interactions. Substantial off-target and toxicity risks remain uncharacterized. For instance, kinase inhibitors like DCLK1-IN-1 may affect structurally similar kinases, and GPCR modulators like COMPOUND 5G could engage unintended receptors, leading to adverse effects.

Our study has made significant progress, but there are also some limitations: (1)Our analysis was limited to individuals of European descent; future studies are needed to verify the applicability of these findings in other ethnic groups. (2)Due to the inability to access additional GWAS outcome data with a larger number of cases and multicenter data, our results may not be generalizable. (3)Although we used a MR study design to mitigate the impact of confounding factors, other potential factors that may affect the results must still be considered. While MR studies are powerful, they can only reveal correlations and cannot definitively establish causal relationships. Therefore, additional experimental studies are needed for validation. (4)The technical limitations and interpretive challenges of single-cell transcriptome sequencing data need to be overcome through further experimental research. Key challenges involve managing data noise, accurately identifying and annotating cell types, normalizing datasets, and selecting suitable statistical methods for differential analysis. Additionally, validation and confirmation steps are essential for ensuring accurate interpretation of gene expression in single-cell studies. (5)Finally, molecular docking serves as a preliminary, hypothesis-generating tool. It primarily assesses binding conformation and theoretical affinity but cannot predict a compound’s pharmacokinetics, toxicological profiles, off-target effects, or overall therapeutic window in living cells or organisms. While this method identified potential drug targets, it does not guarantee their efficacy in clinical settings. Subsequent experimental validation and clinical trials are essential to confirm the therapeutic potential of the identified targets.

Despite these limitations, our integrated multi-omics approach successfully identified novel genetic susceptibility genes and delineated new avenues for the targeted therapy of T1D. Importantly, these findings may serve as testable hypotheses, laying a foundation for future experimental and functional validation studies that further elucidate the pathogenesis of T1D and inform the development of therapeutic strategies.

## 5 Conclusion

Our study identifies several potential disease-modifying agents for future type 1 diabetes (T1D) treatment. The three druggable genes—ELK4, WFS1, and SULT1A2—warrant further investigation to assess their viability as T1D therapeutic targets. These findings delineate pathways for developing more effective T1D therapeutics, which may reduce drug development costs. However, while cross-tissue TWAS analyses offer valuable insights, their extrapolation to clinical practice requires caution. Well-designed clinical trials are essential to validate the therapeutic potential of these targets.

## Data Availability

The original contributions presented in the study are included in the article/**Supplementary Material**. Further inquiries can be directed to the corresponding author.

## Glossary

T1D: Type 1 diabetes
TWAS: Transcriptome-wide association study
GWAS: Genome-wide association study
MR: Mendelian Randomization
SMR: Summary Mendelian Randomization
FUSION: Functional Unit-based Summarized Information Network
DEGs: Differentially expressed genes
TRS: Trait-relevant scores
SNPs: Single nucleotide polymorphisms
IVs: Instrumental variables
IVW: Inverse-variance weighted
CI: Confidence interval
HEIDI: heterogeneity in dependent instruments
sCCA-ACAT: sparse Canonical Correlation Analysis–Aggregated Cauchy Association Test
GO: Gene Ontology
GSEA: Gene set enrichment analysis
BP: biological processes
CC: cellular components
MF: molecular functions
FDR: false discovery rate
