# Supplementary figures and images for: Cross-tissue transcriptome-wide association identify novel T1D susceptibility genes and drug candidates
Source: Front Immunol. 2026 Jan 21;16:1735004. doi: 10.3389/fimmu.2025.1735004 (PMC12867781; doi:10.3389/fimmu.2025.1735004)

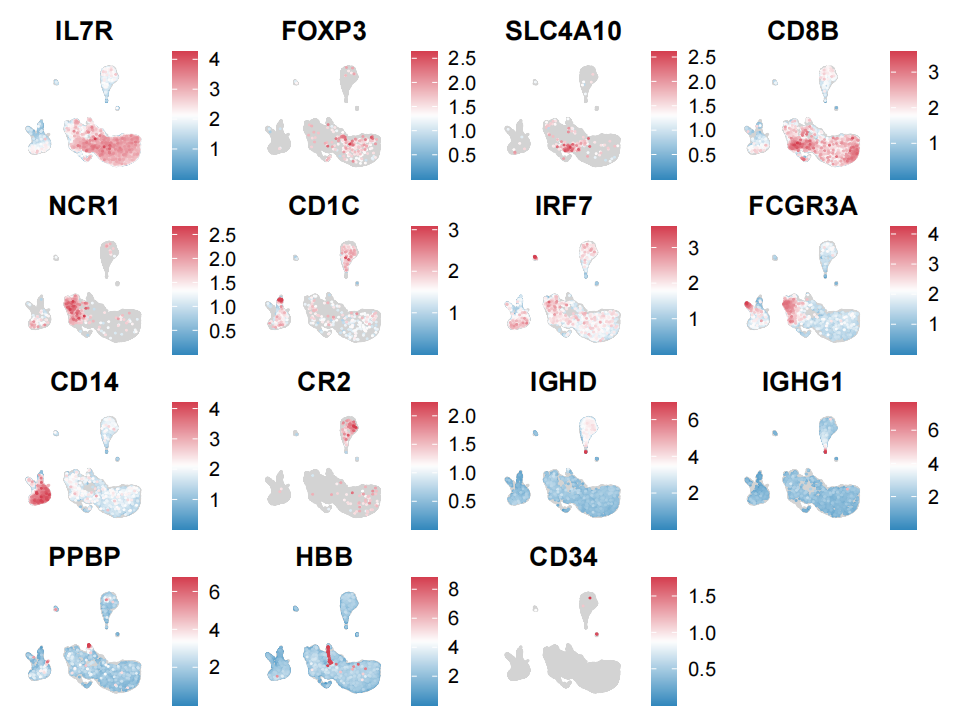

Supplement: Supplementary file 1 [file DataSheet1.zip › Supplementary Figure S2.tif]

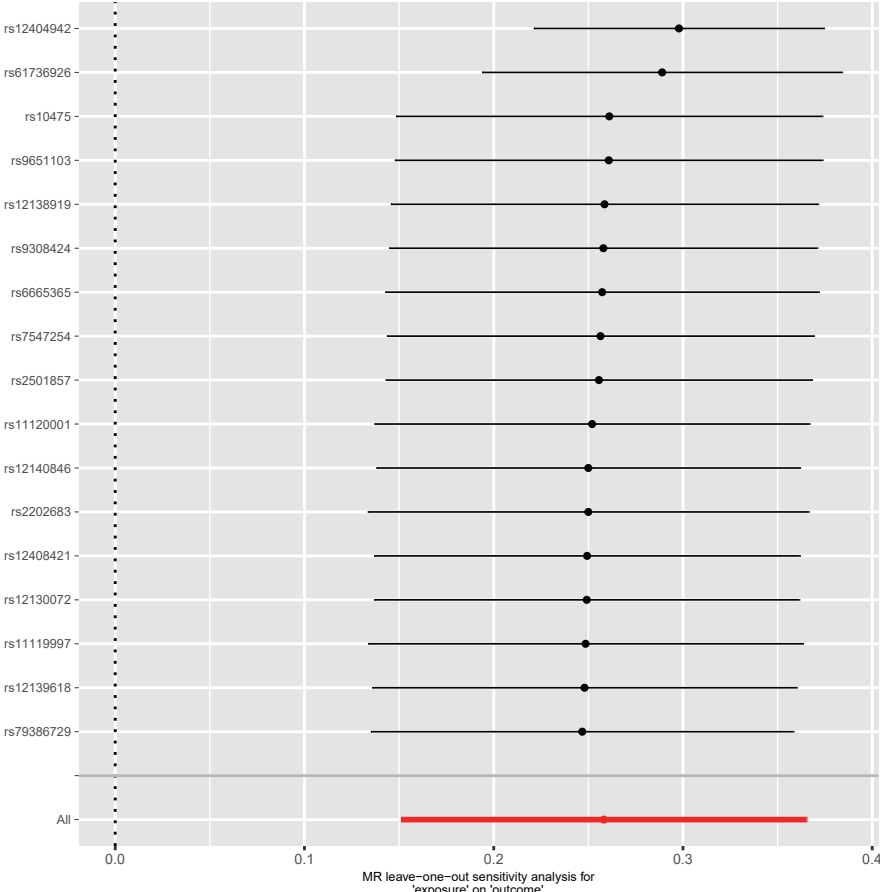

BATF3

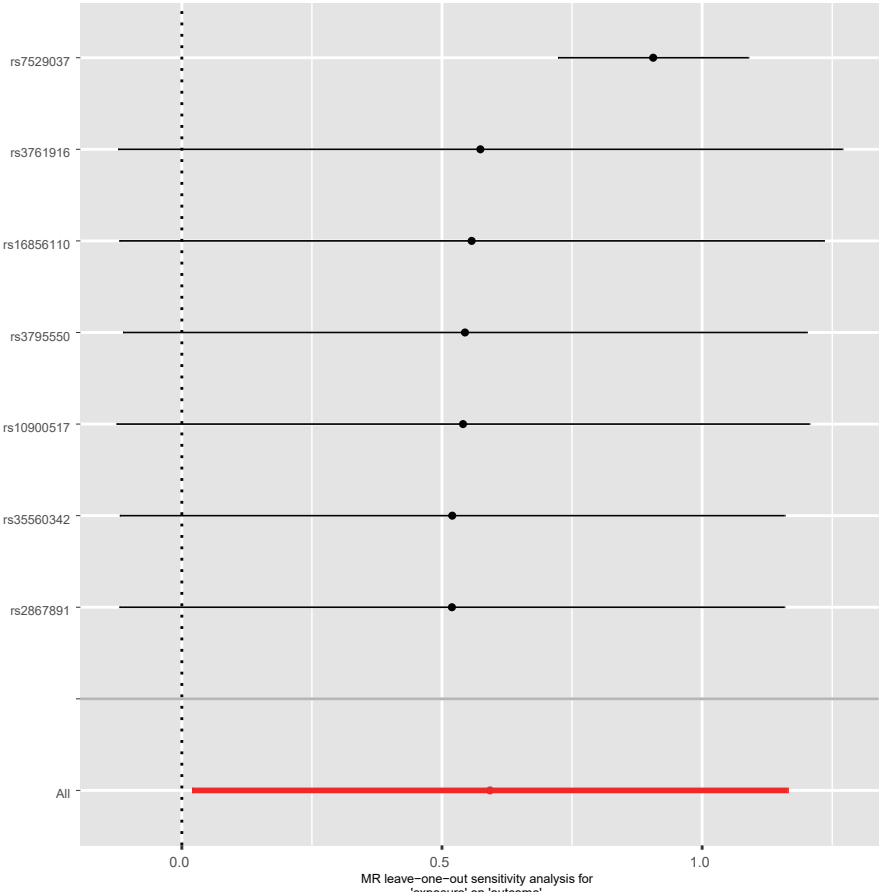

ELK4

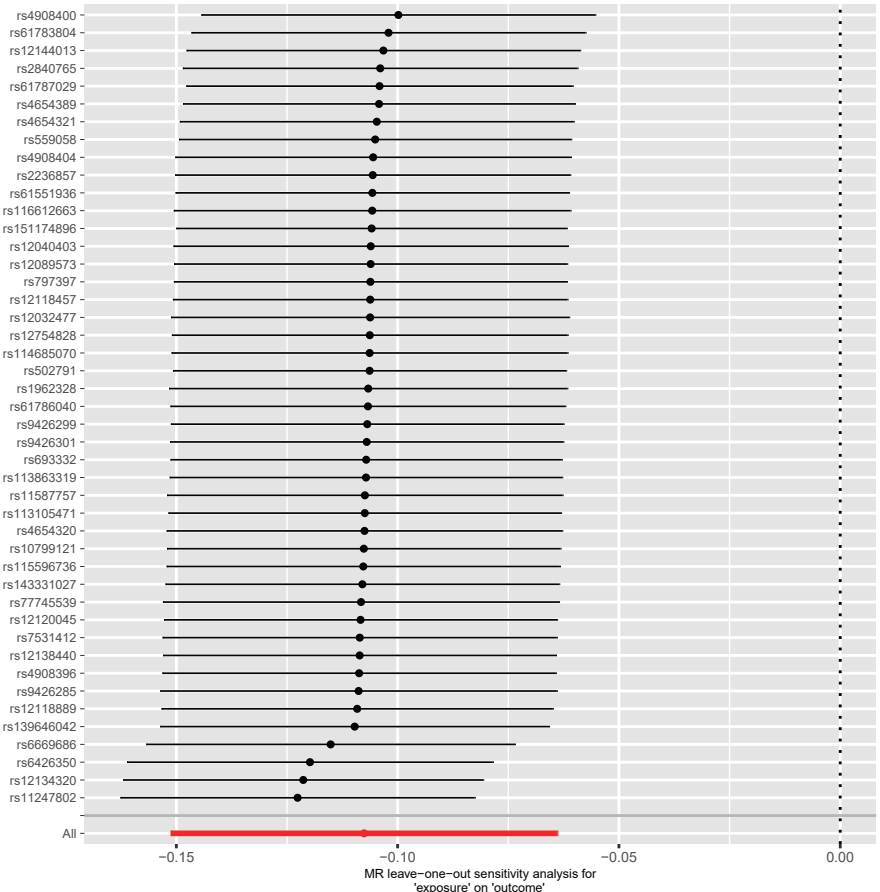

PHACTR4

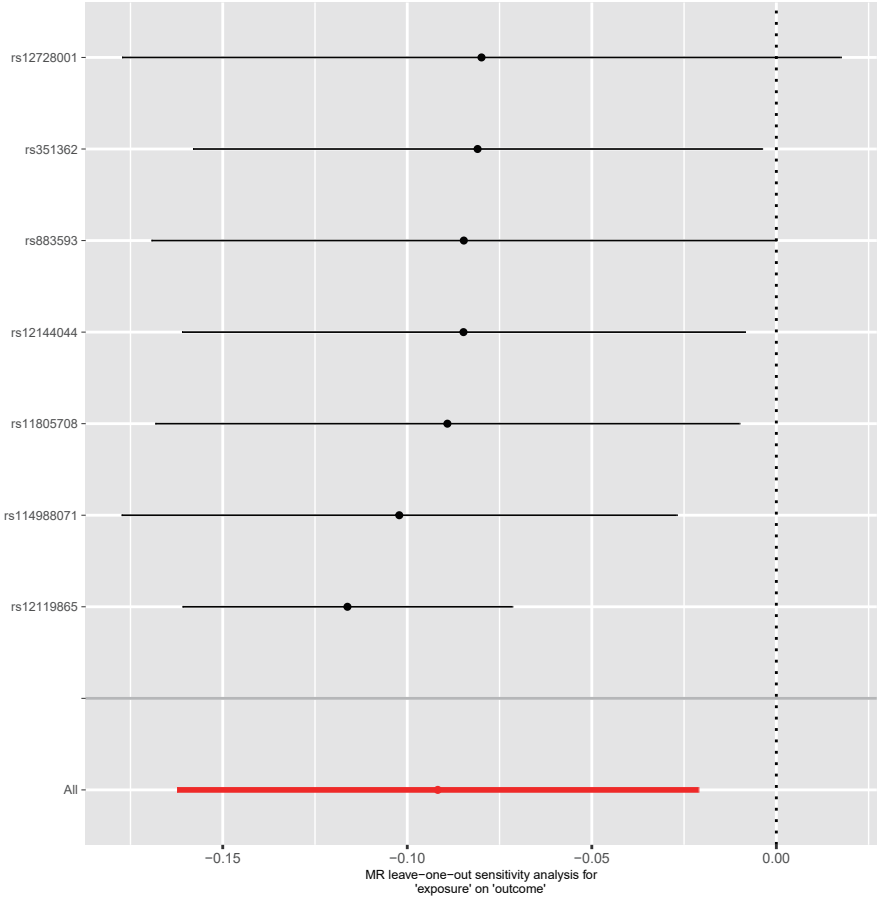

ST7L

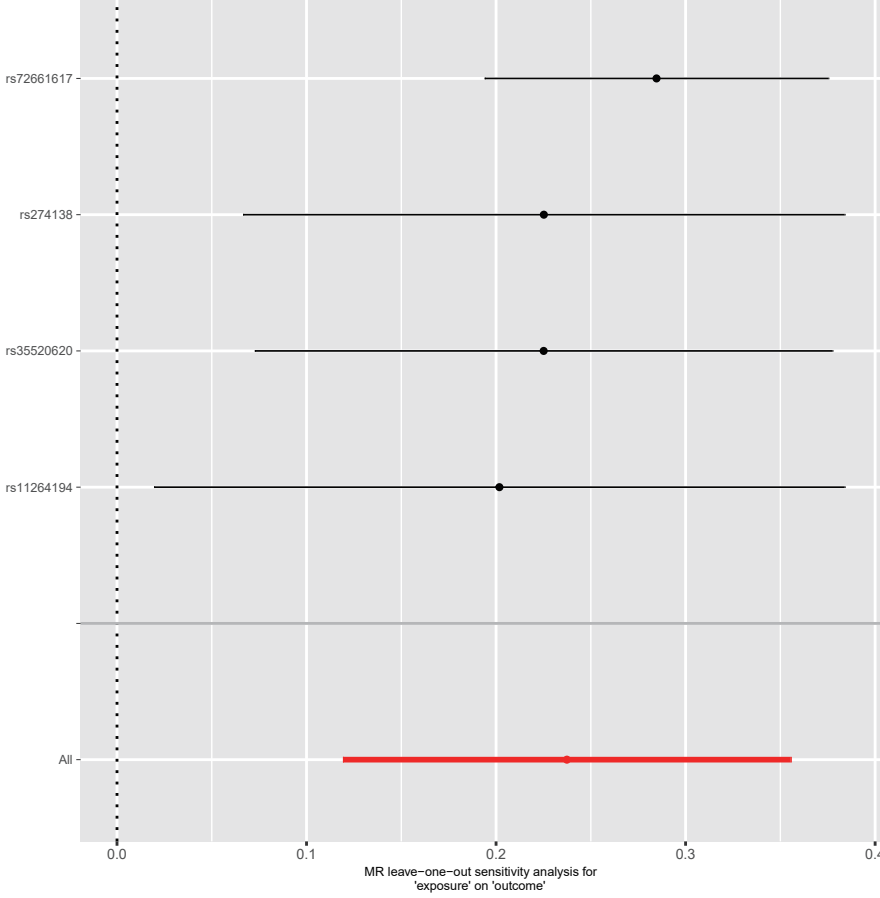

C1orf216

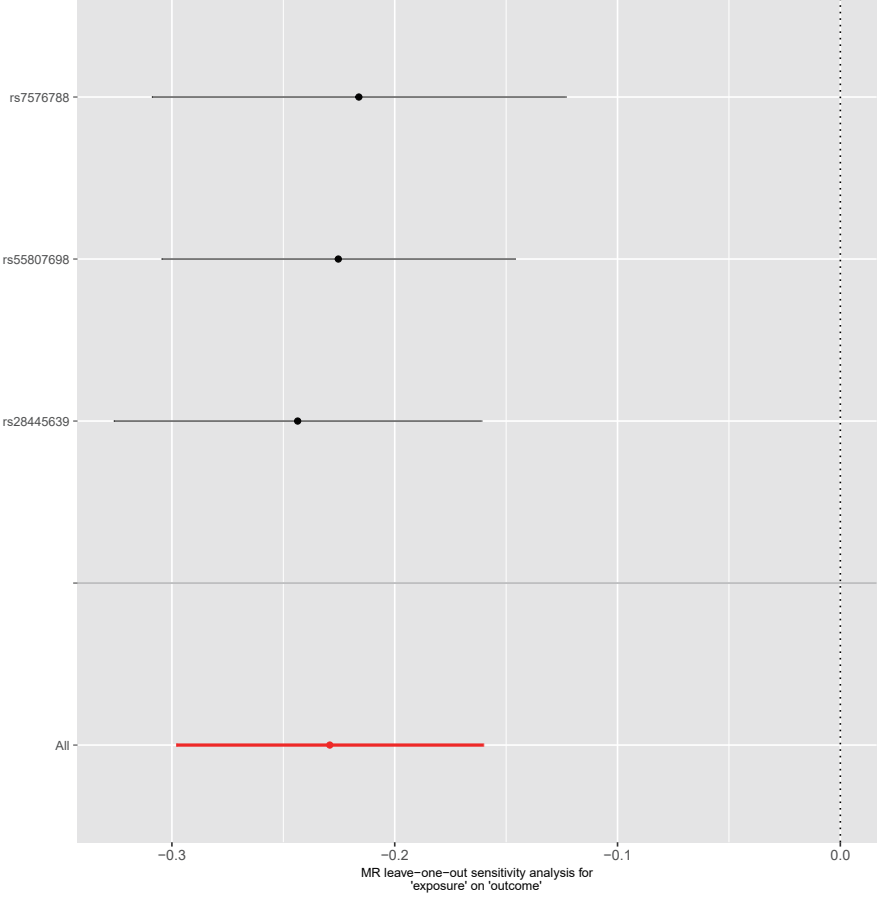

CENPO

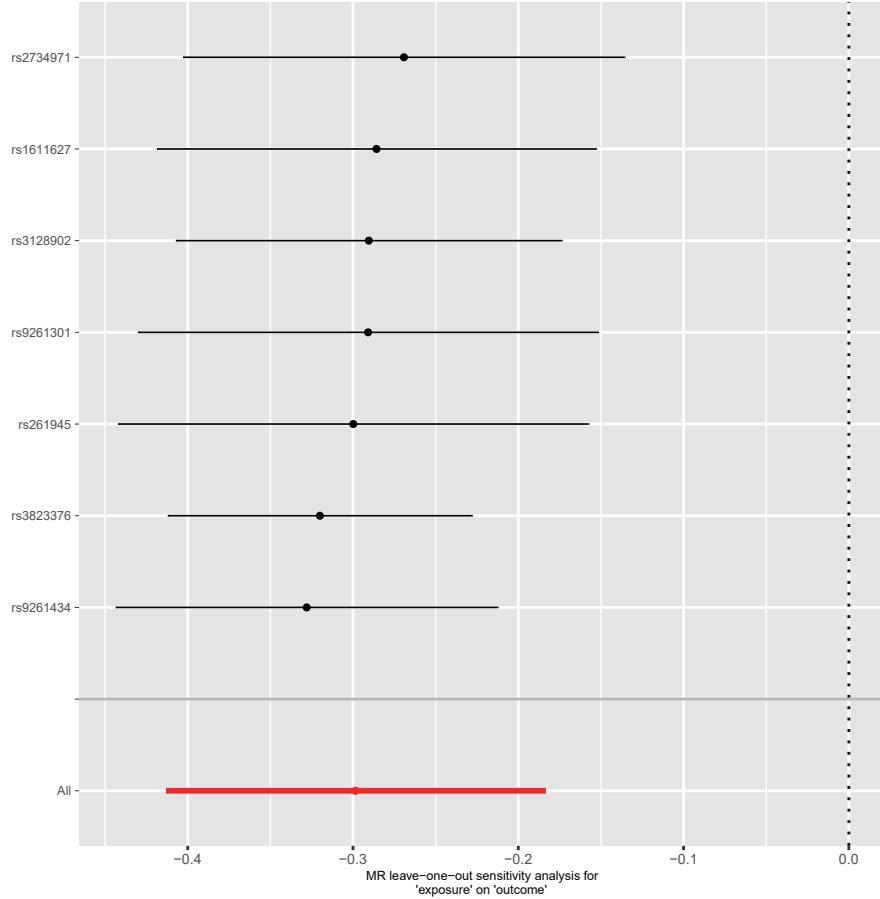

HLA-A

Supplement: Supplementary file 1 [file DataSheet1.zip › Supplementary Figure S1.PDF]
